# Time‐Dependent Bias and Prognostic Confounding in Exposure–Response Analysis of Targeted Therapies: Lessons from Sunitinib in Metastatic Renal Cancer

**DOI:** 10.1002/cpt.70447

**Published:** 2026-08-12

**Authors:** Han Liu, Lena E. Friberg

**Affiliations:** ^1^ Department of Pharmacy Uppsala University Uppsala Sweden

## Abstract

Exposure–response (E–R) analyses of sunitinib in metastatic renal cell carcinoma (mRCC) have supported concentration‐guided dose escalation. However, such analyses may be biased when exposure metrics incorporate post‐baseline dose modifications, time‐varying apparent clearance, or baseline factors that influence both pharmacokinetics and clinical outcomes. We re‐evaluated the E–R relationship of sunitinib in mRCC with explicit assessment of these biases. A population pharmacokinetic analysis of 294 patients from four clinical trials identified time‐dependent declines in apparent clearance for sunitinib and SU12662. E–R analyses were performed in 165 cytokine‐refractory patients treated with the approved 50 mg 4‐weeks‐on/2‐weeks‐off regimen, using time to progression (TTP), overall survival (OS), and tolerability‐related outcomes. Baseline albumin and age were identified as shared covariates of exposure and outcome and incorporated as confounders in multivariable multistate survival models. The apparent positive association between higher exposure and improved TTP and OS was attenuated when exposure was defined using early, baseline‐anchored metrics rather than time‐averaged apparent clearance. It was no longer evident after adjustment for albumin and age. Consistently, simulations with confounders fixed at cohort medians showed overlapping TTP and OS profiles across exposure groups. In contrast, higher exposure remained associated with poorer tolerability, including increased risks of dose reduction and treatment discontinuation driven by adverse‐events. These findings indicate that previously reported positive E–R relationships for sunitinib were largely due to time‐dependent bias and confounding. The results do not support exposure‐guided dose escalation based on total plasma concentrations, but instead suggest the use of therapeutic drug monitoring to identify patients at risk of excessive exposure and toxicity.

**Study Highlights**

**WHAT IS THE CURRENT KNOWLEDGE ON THE TOPIC?**

Apparent associations between higher exposure and longer TTP and OS for sunitinib in mRCC have been used to justify therapeutic drug monitoring (TDM) targets and exposure‐guided dose escalation.

**WHAT QUESTION DID THIS STUDY ADDRESS?**

This study investigated the extent to which time‐dependent biases and baseline confounding distort the apparent E–R relationship in patients with mRCC treated with sunitinib. It systematically evaluated whether the previously reported beneficial survival associations remain valid after mitigating these biases using baseline‐anchored exposure metrics and multivariable multistate survival models.

**WHAT DOES THIS STUDY ADD TO OUR KNOWLEDGE?**

This study suggests that previously reported positive exposure–efficacy associations for sunitinib may largely reflect time‐dependent bias and baseline prognostic confounding, including by albumin and age, rather than a causal benefit of higher exposure. After adjustment, no independent positive exposure–efficacy association was identified, consistent with a weak or plateaued relationship within the exposure range achieved with the standard 50 mg/day 4/2 regimen. In contrast, higher early‐cycle exposure remained strongly associated with poorer tolerability and a higher risk of toxicity‐driven treatment discontinuation.

**HOW MIGHT THIS CHANGE CLINICAL PHARMACOLOGY OR TRANSLATIONAL SCIENCE?**

These findings caution against causal interpretation of apparent exposure–response relationships for targeted therapies without accounting for time‐dependent bias and baseline confounding. In sunitinib‐treated mRCC, they challenge the rationale for exposure‐driven dose escalation and instead support individualized dose de‐escalation strategies, such as TDM‐based identification of patients with excessive early exposure who may benefit from dose reduction, thereby reducing toxicity without compromising long‐term outcomes.

A paradigm shift in oncology drug development toward rigorous dose‐finding and optimization has been strongly advocated by patients, clinicians, and experts^1^, ^2^ and further reinforced by the FDA's Project Optimus initiative.^3^ Central to this shift is the need for robust exposure–response (E–R) evaluation to support dose selection that preserves efficacy while minimizing toxicity.^4^

Although drug exposure is generally expected to influence clinical outcome, observed E–R relationships may be distorted by bias and, if interpreted causally without adequate scrutiny, may mislead dose optimization.^5^, ^6^ Recent methodological work has highlighted key sources of bias in E–R analyses,^7^, ^8^ and case studies in anticancer monoclonal antibodies have shown that such bias can affect both interpretation and clinical decision‐making.^9^, ^10^ In contrast, the extent to which similar bias has influenced E–R analyses of older, widely used targeted therapies, such as tyrosine kinase inhibitors (TKIs), remains insufficiently studied.^11^

TKIs have transformed cancer treatment, with more than 90 agents approved to date.^12^ Their E–R analyses typically examine associations between drug exposure and time‐to‐event outcomes such as time to progression (TTP) and overall survival (OS), with exposure commonly characterized using total (bound plus unbound) plasma concentrations.^13^ Early‐generation TKIs may be particularly vulnerable to bias for at least two reasons. First, dose selection for many of these agents was established in phase I studies through identification of the maximum tolerated dose (MTD), which was then carried forward as the only dose tested in pivotal phase II and III trials, whereas formal dose‐randomized studies were rarely conducted.^14^ Consequently, many E–R analyses were performed within a single‐dose setting, such that interindividual differences in exposure mainly reflected pharmacokinetics (PK) variability rather than randomized dose differences. Therefore, confounding may arise when exposure‐related factors are also prognostic for clinical outcomes. Second, bias may be introduced by the exposure metric itself. Many E–R analyses use exposure summaries averaged over the observed treatment duration, such as the time‐averaged area under the concentration–time curve (AUC), which, in simplified terms, reflects dose relative to apparent clearance. When dose or apparent clearance changes during follow‐up, such metrics can introduce time‐dependent bias.^15^ For instance, if the apparent clearance declines over time, patients who remain on treatment longer will, by construction, appear to have higher average exposure, thereby generating an artifactually positive association between exposure and survival. This mechanism was previously characterized for nivolumab, for which first‐dose rather than steady‐state PK metrics were proposed to better reflect the underlying E–R relationship.^16^ For TKIs, despite time‐varying apparent clearance being reported for several agents,^17^, ^18^ its implications for bias in E–R analyses have not been systematically evaluated.

In addition, when exposure summaries incorporate the average administered dose in settings where dose reductions are common, patients with longer survival have more opportunity to undergo dose reductions. They may therefore appear to have lower average exposure, producing an attenuated or even negative association. This issue has been highlighted for lenvatinib,^19^ but is broadly relevant for TKIs, for which dose interruptions and reductions due to toxicity are common.

Sunitinib is a multi‐targeted vascular endothelial growth factor receptor (VEGFR) inhibitor approved in 2006 for several malignancies, including metastatic renal cell carcinoma (mRCC).^20^ It is metabolized primarily by CYP3A4 to the active metabolite SU12662, and both sunitinib and SU12662 are eliminated predominantly via biliary and fecal routes, with minimal renal excretion. Both compounds are highly protein‐bound in plasma (~95% and ~90%, respectively), mainly to albumin.^21^ In mRCC, the approved dose of 50 mg once daily on a 4‐weeks‐on/2‐weeks‐off schedule was established as the MTD in a phase I study,^22^ and was subsequently evaluated in two pivotal phase II trials that supported regulatory approval.^23^, ^24^ Although the MTD was defined based on short‐term toxicity, patients often remain on treatment for months, and toxicities frequently necessitate dose modification; dose reductions have been reported in approximately 16–49% of patients and discontinuation due to adverse events (AEs) in 3–16%.^23^, ^24^, ^25^, ^26^

Notably, prior post hoc analyses of sunitinib in mRCC reported positive univariable associations between higher total exposure and improved TTP and OS under the standard 50 mg 4/2 regimen,^27^, ^28^ findings that helped motivate proposals for concentration‐guided dose escalation in patients with apparently suboptimal exposure.^13^, ^29^, ^30^ However, these analyses were conducted in a setting in which both time‐dependent bias and biological confounding are plausible. Sunitinib therefore provides a clinically relevant case study for examining how such bias may influence apparent E–R relationships for TKIs.

In the present study, we evaluated the extent to which dose reduction, time‐varying apparent clearance, and confounding bias the apparent E–R relationship of sunitinib in mRCC. Specifically, we characterized dose‐reduction patterns over time, assessed time‐varying apparent clearance, and identified baseline covariates that could influence both exposure and clinical outcomes. We then accounted for these factors when estimating the association between total drug exposure and survival outcomes. Finally, to provide a more complete assessment of benefit–risk, we also evaluated the relationship between exposure and tolerability‐related outcomes.

## METHODS

### Patients and data

De‐identified individual participant data were obtained through the Vivli data sharing platform from four completed clinical trials of sunitinib in mRCC: study 1006 (NCT00077974), study 014 (NCT00054886), study 1034 (NCT00083889), and study 1065 (NCT00267748).^23^, ^24^, ^25^, ^31^ All studies were conducted in accordance with the Declaration of Helsinki and Good Clinical Practice, with institutional ethics approval obtained for the original protocols. Studies 1006 and 014 were pivotal phase II trials in cytokine‐refractory mRCC, whereas Studies 1034 and 1065 enrolled treatment‐naïve patients (**Table** **1**).

**Table 1:** Summary of clinical studies included in this study

Study	Study 1006	Study 014	Study 1034	Study 1065
NCT	NCT00077974	NCT00054886	NCT00083889	NCT00267748
Description	Phase II, pivotal nonrandomized study	Phase II, pivotal nonrandomized study	Phase III, randomized study of sunitinib vs. IFNalfa	Phase II, randomized study
Population	Cytokine‐refractory patients		Treatment naive	
Dosing schedule	50 mg q.d. (4/2)	50 mg q.d. (4/2)	50 mg q.d. (4/2)	50 mg q.d. (4/2) or 37.5 mg q.d. (cdd)
Patients enrolled on 50 mg q.d. (4/2)	106	63	375	146
Median TTP follow‐up	7.8 months	5.8 months	9.7 months	5.5 months
Longest TTP follow‐up	46.3 months	15.9 months	45.1 months	26.0 months
Median OS follow‐up	21.9 months	12.4 months	23.7 months	21.4 months
Longest OS follow‐up	58.5 months	20.3 months	47.2 months	36.1 months
Patients included in PK analysis	105	60	42	88
PK sampling time	Cycle 1: day 14, 28 Cycle 2–4: day 1, 28 Cycle 5: day 1	Cycle 1: day 7, 14, 21, 28, 35 Cycle 2–3: day 1, 7, 14, 21, 28 Cycle 4–5: day 1, 14, 28 Cycle 6: day 1	Cycle 1–4: day 1, 29	Cycle 1: day 29 Cycle 2: day 1
Tumor assessment schedule	Every 6 weeks for 4 cycles, and then every 12 weeks			Every 8 weeks

cdd, continuous daily dosing; IFN, interferon; PK, pharmacokinetic; q.d., once daily.

Baseline covariates included demographic, clinical, and laboratory or organ function variables, including age, sex, body weight, ECOG performance status, tumor size, albumin, hemoglobin, serum creatinine, and liver enzymes. The full covariate list and correlation structure are provided in **Figure** **S1**. Baseline covariates with missingness below 10% were included in covariate analysis, and missing values were handled using median imputation.

The population PK analysis included 294 patients with PK observations under the 50 mg 4/2 regimen pooled across the four studies. PK samples were collected at trough during the 4‐week treatment period and at the end of the 2‐week break before the next cycle in all patients; a subset also had samples collected 1 week after the off‐treatment period (**Table** **1**). Sampling was extended to 5 cycles in Study 014, 4 cycles in Studies 1006 and 1034, and was collected only during the first cycle in Study 1065. Total (bound plus unbound) plasma concentrations of sunitinib and SU12662 were quantified using validated LC–MS/MS assays.^22^, ^32^ The temporal distribution of concentration measurements, stratified by study, is visualized in **Figure** **S2**.

Local investigators assessed tumor response according to prespecified schedules using RECIST v1.0, from which TTP was derived. Median follow‐up for TTP and OS is summarized in **Table** **1**. Exposure–response analyses of TTP and OS were restricted to the cytokine‐refractory mRCC cohort from Studies 1006 and 014, thereby using the same clinical dataset as the prior post hoc analyses that reported positive exposure–outcome associations under the standard 50 mg 4/2 regimen.^27^, ^28^ Across studies 1006 and 014, 169 patients received at least one dose of sunitinib 50 mg (4/2), with median treatment durations of 264 days and 235 days, respectively (**Table** **1**). Four patients were excluded as incomplete dose‐interruption records precluded reliable reconstruction of dosing histories and exposure estimation, leaving 165 patients in the E–R analysis set.

### Model for pharmacokinetic data

A population PK analysis was conducted to characterize plasma concentrations of sunitinib and SU12662, considering actual dosing histories. The structural model was based on a two‐compartment framework with first‐order absorption and elimination for both compounds.^33^ The first‐order absorption rate constants (k_a_) were fixed to literature values due to the absence of absorption‐phase data.^33^ Apparent clearances and apparent volumes of distribution were estimated based on data obtained following oral administration with $F_{\text{sunitinib}}$ fixed to 1. The fraction of sunitinib metabolized to SU12662 ($f_{m}$) was fixed to 0.21 ($F_{\mathrm{SU}12662}$ = $F_{\text{sunitinib}}\cdot f_{m}$).^33^ Correlations in IIV of the PK parameters for sunitinib and SU12662 were captured using an omega block structure, reflecting their shared disposition pathways. The PK parameters were assessed for potential time‐ and baseline‐covariate effects. Further details of the model development and covariate analysis are provided in the **Supplementary Materials**.

### Model for time‐to‐event outcomes

Survival outcomes of interest, including TTP, OS, and treatment discontinuation due to AEs (reason for discontinuation as indicated by the source dataset), were jointly described using a multistate survival model.^34^, ^35^, ^36^ The model consists of six states with the following definitions:

- S1: Stable (SD: stable disease).
- S2: Response (PR: partial response or CR: complete response).
- S3: Progression (PD: progressive disease).
- S4: Discontinuation due to AEs (+28 days after the last date of drug taken).
- S5: Discontinuation due to other reasons (+28 days after the last date of drug taken, including sponsor decision, protocol violation, loss to follow‐up, or non‐RECIST‐confirmed progression).
- S6: Death.

**Figure 1:**
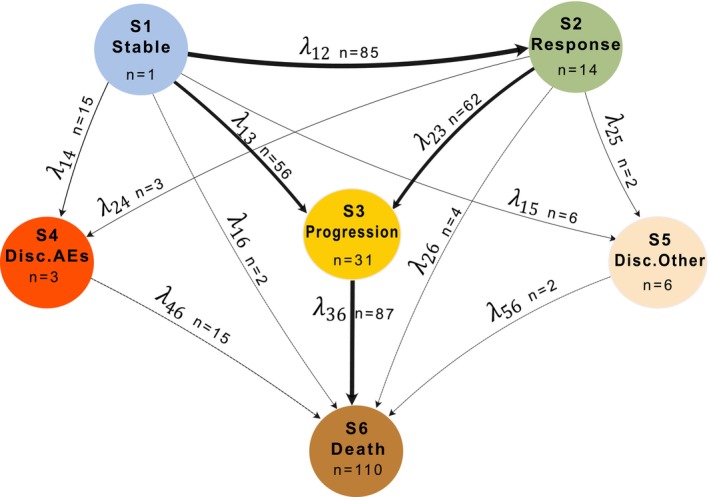
Transition diagram for the multistate model describing disease course and treatment outcomes. Circles represent health states: S1 (Stable), S2 (Response), S3 (Progression), S4 (Discontinuation due to adverse events), S5 (Discontinuation due to other reasons), and S6 (Death). Arrows indicate transitions between states. The notation $\lambda_{\mathrm{ij}}$ denotes the transition hazard from state 𝑖 to state 𝑗 estimated as model parameters. Numbers shown on the arrows correspond to the observed number of transitions in the exposure–response analysis dataset (*n* = 165 individuals). The numbers shown on the state circle represent the observed number of patients censored within each state at the end of the study.

The state and transition diagram is shown in **Figure** **1**. All patients are assumed to be in S1 at the time of the first dose. The probability of occupying state *n* was modeled as the amount in the compartment for each state, quantified by transition hazards $\lambda_{\mathrm{ij}}$ from state 𝑖 to state 𝑗, estimated as model parameters. Baseline covariates and time were tested as predictors of the state transition hazards and hazard of dose reduction. Further technical details, including how clinical endpoints are derived from the transition records and covariate analysis, are summarized in the **Supplementary Materials**.

The time to first dose reduction (50 to 37.5 mg/day) was characterized using a parametric time‐to‐event model.

### Exposure metrics

Systemic exposure was quantified using the steady‐state area under the concentration–time curve (${\mathrm{AUC}}_{\mathrm{ss}}$) for the combined concentrations of sunitinib and SU12662, which are equipotent *in vivo* ^28^, ^37^:

(1) $${\mathrm{AUC}}_{\mathrm{ss}}=\frac{\text{Dose}}{{\mathrm{CL}}_{\text{sunitinib}}/F_{\text{sunitinib}}}+\frac{\text{Dose}}{{\mathrm{CL}}_{\mathrm{SU}12662}/F_{\mathrm{SU}12662}}$$

Here, ${\mathrm{CL}}_{\text{sunitinib}}$, ${\mathrm{CL}}_{\mathrm{SU}12662}$, $F_{\text{sunitinib}}$, and $F_{\mathrm{SU}12662}$ represent individual pharmacokinetic parameters (Empirical Bayes Estimates, EBEs) based on the final population PK models.

Three exposure metrics (${\mathrm{AUC}}_{\mathrm{ss}}$) were derived:

- ${\mathrm{AUC}}_{\mathrm{ss},{\text{DOSE}}_{50},{\mathrm{CL}}_{\text{cycle}1}}$ – calculated from the initial 50 mg dose and cycle 1 apparent clearance, serving as the primary exposure metric not subject to time‐dependent bias.
- ${\mathrm{AUC}}_{\mathrm{ss},{\text{DOSE}}_{\mathrm{ave}},{\mathrm{CL}}_{\text{cycle}1}}$ – calculated from the individual time‐averaged dose and apparent clearance based on cycle 1 PK data, reflecting the potential impact of dose reductions.
- ${\mathrm{AUC}}_{\mathrm{ss},{\text{DOSE}}_{50},{\mathrm{CL}}_{\mathrm{ave}}}$ – calculated from the initial assigned dose of 50 mg and time‐averaged apparent clearance, reflecting the potential impact of time‐varying clearance.

Alternative exposure metrics were evaluated in sensitivity analyses of the E–R relationships, including unbound exposure, calculated as total exposure weighted by the unbound fractions of sunitinib and SU12662 (i.e., ${\mathrm{AUC}}_{\mathrm{ss},\text{sunitinib}}\cdot 0.1+{\mathrm{AUC}}_{\mathrm{ss},\mathrm{SU}12662}\cdot 0.05$). Given that previous concentration‐guided therapeutic drug monitoring (TDM) studies proposed steady‐state trough concentration ($C_{\text{trough},\mathrm{ss}}$) thresholds of 50 or 60.75 ng/mL for efficacy,^38^, ^39^, ^40^ $C_{\text{trough},\mathrm{ss}}$ was also evaluated as a categorical predictor of survival outcomes, dichotomized as below vs. at or above each threshold.

### Exposure–response analysis for efficacy outcomes

Associations between various exposure metrics and TTP and OS were examined using Kaplan–Meier (K–M) curves and log‐rank tests, stratified by median exposure. Exposure–efficacy relationships were further assessed for significance in the multistate model by testing exposure as a predictor of transition hazards. Initially, univariate and thereafter multivariable models adjusted for baseline confounders, which were defined as baseline covariates found to impact both PK and survival outcomes, were tested.

To visualize Exposure–efficacy associations after adjustment for baseline confounding, we simulated survival outcomes using the point estimates from the final multivariable multistate survival model. Simulations were performed on the observed multistate dataset, in which each patient's baseline confounders were replaced with the cohort median (i.e., identified confounding factors were set to the population median for all individuals). Survival curves were then generated for patients stratified by the median exposure representing the expected TTP and OS under a common baseline prognostic profile.

### Exposure–response analysis for tolerability‐related outcomes

Exposure was evaluated as a predictor of (i) time to treatment discontinuation due to AEs using the multistate survival model, and (ii) time to first dose reduction using a separate parametric hazard model.

### Software and model evaluation

Population PK, multistate survival, parametric hazard, and proportional odds models were developed using NONMEM (version 7.5)^41^ and Perl‐speaks‐NONMEM (PsN, version 5.2.0).^42^ Model selection was guided by likelihood ratio tests and simulation‐based diagnostics. Details on the software and estimation algorithms for the PK and multistate models are provided in the **Supplementary Materials**.

## RESULTS

### Time‐dependent pharmacokinetics and dose intensity

**Figure 2:**
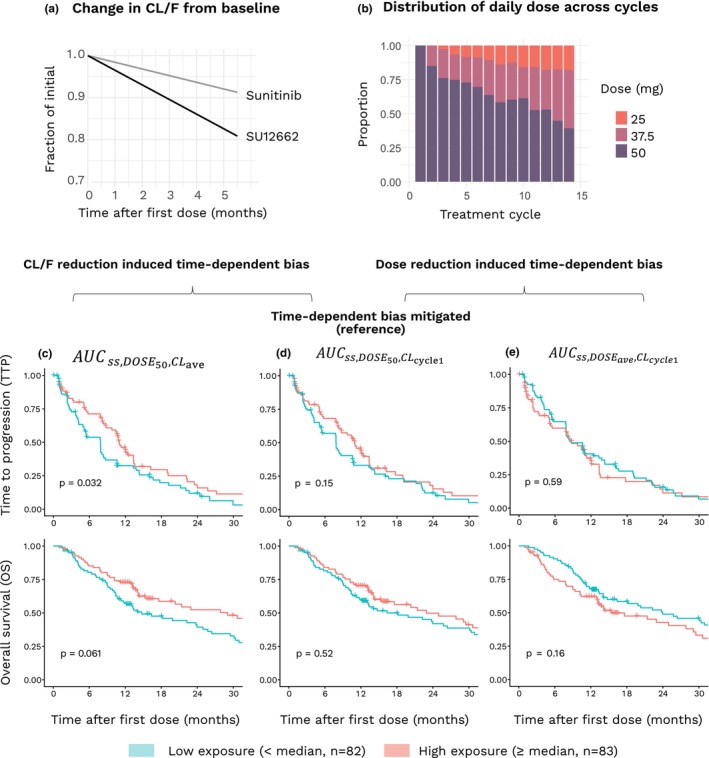
Time‐varying apparent clearance and dose and their impact on the apparent exposure–efficacy relationship. (**a**) Linear decrease of cycle 1 apparent clearance of sunitinib and SU12662 over the first five treatment cycles in a typical individual as a fraction of the baseline value, as identified by the developed population pharmacokinetic model. Note that the estimation was based on PK data from the first five treatment cycles and may therefore not be accurately extrapolated to later cycles. (**b**) Distribution of daily dose across treatment cycles (25, 37.5, or 50 mg). Proportions are calculated as the number of patients receiving each dose level divided by the total number of patients remaining on treatment. (**c–e**) Kaplan–Meier (K–M) plots of TTP and OS stratified on median split of exposure, calculated using (**c**) ${\mathrm{AUC}}_{\mathrm{ss},{\text{DOSE}}_{50},{\mathrm{CL}}_{\mathrm{ave}}}$, (**d**) ${\mathrm{AUC}}_{\mathrm{ss},{\text{DOSE}}_{50},{\mathrm{CL}}_{\text{cycle}1}}$, and (**e**) ${\mathrm{AUC}}_{\mathrm{ss},{\text{DOSE}}_{\mathrm{ave}},{\mathrm{CL}}_{\text{cycle}1}}$. Red represents individuals above or at the median, and turquoise represents those below the median. *P*‐values are from the log‐rank test of K–M survival curves between groups.

Both sunitinib and SU12662 were well described by two‐compartment models with first‐order absorption and linear elimination. A linear decrease in apparent clearance and volume of distribution over time for both the parent and the metabolite significantly improved the model fit (ΔOFV = −197). The estimated time‐varying trends corresponded to decreases of 8.8% and 19.2% in apparent clearance by the end of cycle 4 (**Figure** **2** **a**) and decreases of 4.0% and 30.0% in apparent volume of distribution over the same period in a typical individual. Further details on the final PK model structure, parameter estimates, and diagnostics are provided in the **Supplementary Materials**. In an exploratory comparison, the individual magnitude of apparent clearance decline did not differ significantly between responders and non‐responders. However, η‐shrinkage for the IIV in the clearance‐decline parameters was high (45.9% for sunitinib and 60.9% for SU12662) limiting the reliability of comparisons based on individual parameter estimates. Together with the sparse longitudinal PK sampling, these findings should therefore be interpreted cautiously.

Dose reductions from the initial 50 mg/day were frequent, occurring in 63 of 165 patients (38.2%) in the E–R analysis dataset. Among patients who remained on treatment, the distribution of administered doses shifted over time, with a declining proportion maintained at 50 mg and increasing proportions receiving 37.5 mg or 25 mg across cycles (**Figure** **2** **b**), indicating that patients treated for longer durations tended to receive lower average dose intensity.

### Exposure metrics

In the E–R analysis dataset (*n* = 165), individual ${\mathrm{AUC}}_{\mathrm{ss},{\text{DOSE}}_{50},{\mathrm{CL}}_{\text{Cycle}1}}$ values exhibited a right‐skewed distribution, with a median (IQR) of 2.19 (1.62–2.99) μg·h/mL and a range of 0.56–5.20 μg·h/mL. The corresponding $C_{\text{trough},\mathrm{ss},{\text{DOSE}}_{50},{\mathrm{CL}}_{\text{Cycle}1}}$ values were highly correlated with ${\mathrm{AUC}}_{\mathrm{ss},{\text{DOSE}}_{50},{\mathrm{CL}}_{\text{Cycle}1}}$ (*r* = 0.99), with a median (IQR) of 81.8 (60.5–116) and a range of 20.8–194.1 ng/mL (**Figures** **S5** **–S** **7**).

Relative to ${\mathrm{AUC}}_{\mathrm{ss},{\text{DOSE}}_{50},{\mathrm{CL}}_{\text{Cycle}1}}$, the median ${\mathrm{AUC}}_{\mathrm{ss},{\text{DOSE}}_{50},{\mathrm{CL}}_{\mathrm{ave}}}$ was slightly higher (2.27 vs. 2.19 μg·h/mL) due to lower average clearance than cycle 1 clearance, whereas the median ${\mathrm{AUC}}_{\mathrm{ss},{\text{DOSE}}_{\mathrm{ave}},{\mathrm{CL}}_{\text{cycle}1}}$ was lower (1.38 vs. 2.19 μg·h/mL) due to lower average doses than the initial 50 mg dose. Descriptive statistics for exposure metrics, stratified by parent drug vs. metabolite, are provided in **Table** **S3**.

### Impact of time‐dependent bias on exposure–efficacy associations

Apparent exposure–efficacy associations in cytokine‐refractory patients (*n* = 165) differed according to the definition of exposure (**Figure** **2** **c–e**). When exposure was summarized as ${\mathrm{AUC}}_{\mathrm{ss},{\text{DOSE}}_{50},{\mathrm{CL}}_{\mathrm{ave}}}$(panel c), higher exposures were associated with longer TTP (log‐rank *P* = 0.032) and a close‐to‐significant difference in OS (*P* = 0.061). Using the baseline‐anchored metric ${\mathrm{AUC}}_{\mathrm{ss},{\text{DOSE}}_{50},{\mathrm{CL}}_{\text{cycle}1}}$ (panel d), the difference was attenuated (TTP *P* = 0.15; OS *P* = 0.52), although the trend remained directionally consistent with improved outcomes at higher exposures. In contrast, using ${\mathrm{AUC}}_{\mathrm{ss},{\text{DOSE}}_{\mathrm{ave}},{\mathrm{CL}}_{\text{Cycle}1}}$ (panel e), there was no evidence supporting a difference for either TTP (*P* = 0.59) or OS (*P* = 0.16) as the trend flipped to longer survival for patients in the lower exposure group.

Given the sensitivity of these associations to exposure definitions based on time‐averaged quantities, ${\mathrm{AUC}}_{\mathrm{ss},{\text{DOSE}}_{50},{\mathrm{CL}}_{\text{cycle}1}}$ was carried forward as the primary exposure metric for subsequent analyses.

### Baseline age and albumin impacted both PK and clinical outcomes

**Figure 3:**
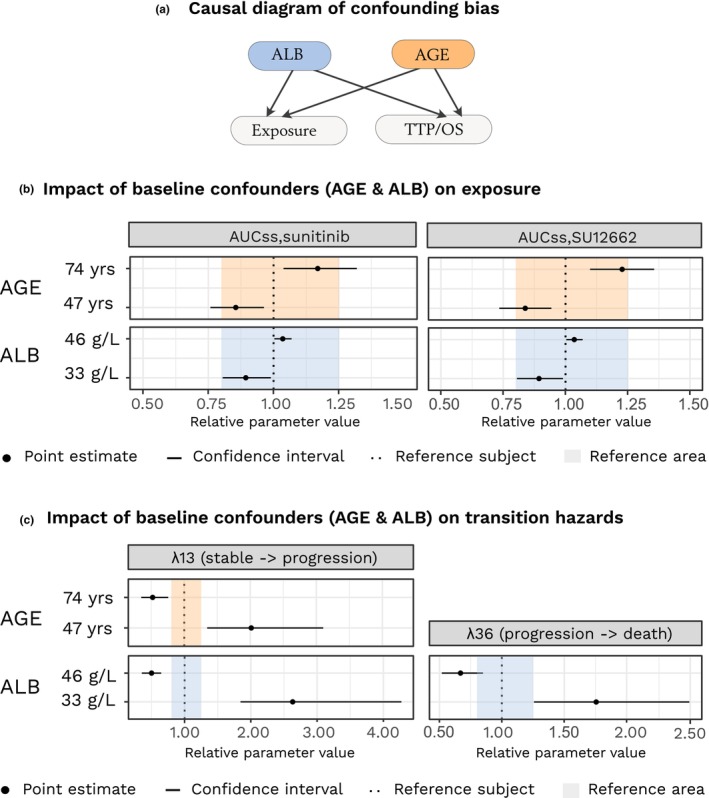
Confounding in the sunitinib exposure–efficacy relationship. (**a**) Directed acyclic graph (DAG) illustrating how baseline albumin (ALB) and age (AGE) influence both drug exposure and clinical outcomes (time to progression [TTP] and overall survival [OS]), thereby acting as confounders in exposure–efficacy analyses. (**b**, **c**) Forest plot illustrating the effects of AGE and ALB on steady‐state AUC for sunitinib and SU12662, based on the final population PK model (**b**), and the effects of AGE and ALB on transition hazards $\lambda_{13}$ (stable disease to progression) and $\lambda_{36}$ (progression to death), based on the final multistate model (**c**). Covariate effects are shown relative to a typical reference subject. For both AGE and ALB, the 10th and 90th percentiles of the observed distribution were selected. Closed circles represent the predicted relative change from the reference subject, and bars indicate 90% confidence intervals reflecting parameter uncertainty, based on 200 parameter vectors sampled from the parameter uncertainty distribution.

Baseline albumin and age were identified as key covariates with shared influence on both systemic exposure and clinical outcomes, suggesting potential confounding of naïve exposure–efficacy associations (**Figure** **3** **a**).

### Impact on pharmacokinetics and exposure

In the population PK model, baseline albumin and age were significant predictors of baseline apparent clearance for both sunitinib and SU12662 (**Figure** **3** **b**). Higher albumin level was associated with lower apparent clearance and thus higher steady‐state AUC; a 10% increase in albumin corresponded to a 4.7% increase in AUC for both compounds. Older age was likewise associated with lower apparent clearance and higher exposure, with a 10‐year increase corresponding to reductions of 5.7% and 7.6% in apparent clearance for sunitinib and SU12662, respectively. Additional PK covariates included sex and body weight; the estimated effect sizes are summarized in **Table** **S2**.

### Impact on time to progression and overall survival

**Figure 4:**
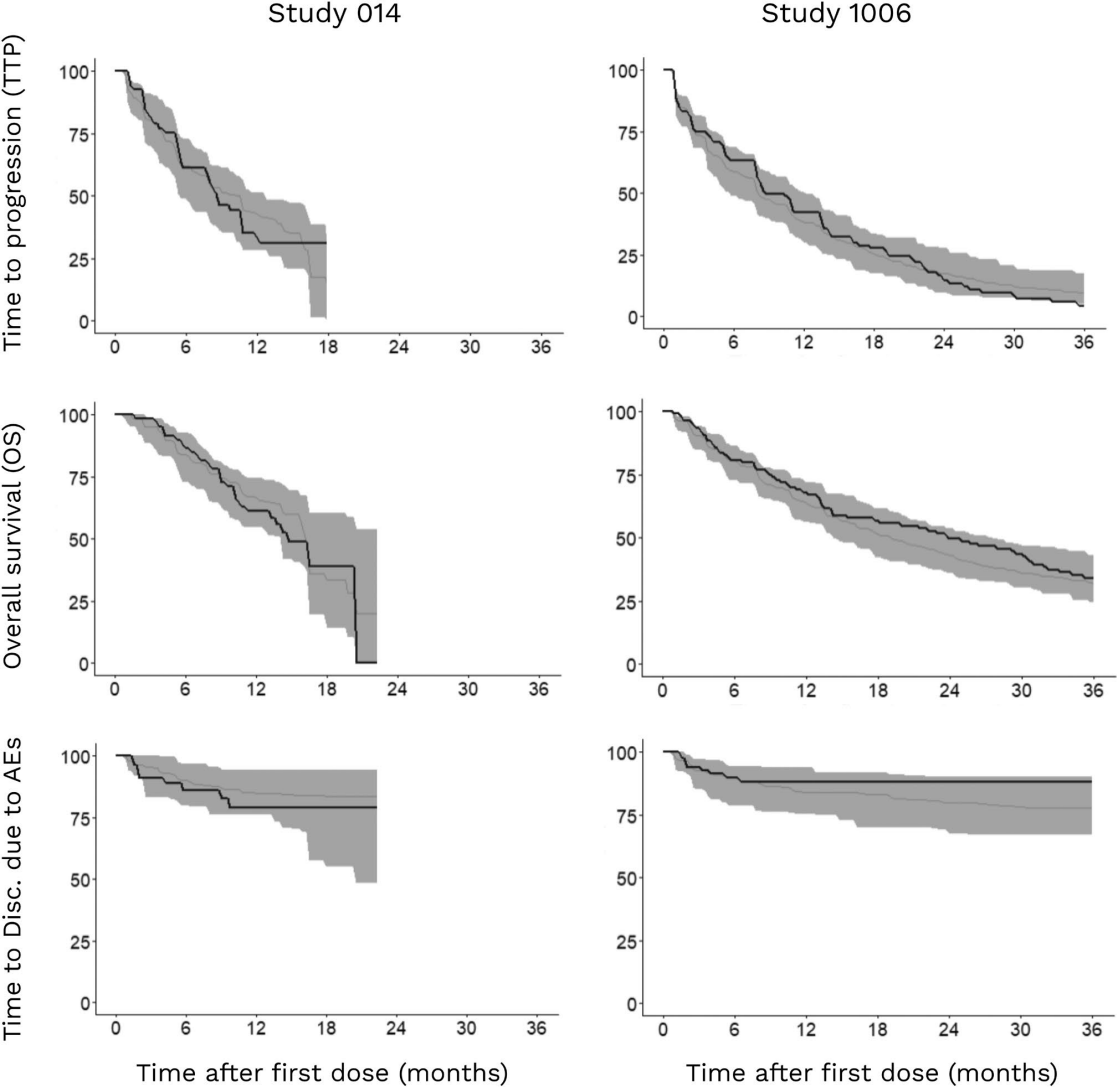
Simulation‐based diagnostic of the final multistate model. Visual predictive checks (VPCs) of the Kaplan–Meier curves for time to progression (TTP), overall survival (OS), and time to treatment discontinuation due to adverse events, stratified by study, in patients treated with sunitinib. Black solid lines represent the observed data, gray solid lines represent the model‐predicted medians, and gray shaded areas indicate 95% CIs from 100 simulations.

In the multistate survival model developed in the cytokine‐refractory cohort (*n* = 165), Albumin and age were additionally associated with clinical outcomes. Lower albumin was associated with a higher hazard of $\lambda_{13}$ (stable disease to progression) and $\lambda_{36}$ (progression to death), with a *P*‐value <0.001 (ΔOFV = −18.3 and − 14.4, respectively) in univariate analysis (**Figure** **3** **c**). Older age was associated with a lower $\lambda_{13}$ (stable disease to progression) with *P* < 0.001 (ΔOFV = −11.72) (**Figure** **3** **c**). Additional predictors identified for TTP and OS beyond albumin and age are summarized in **Table** **S4**. The visual predictive checks in **Figure** **4** showed that the model adequately described both TTP and OS across the included studies.

### Impact of confounding bias on exposure–efficacy associations

The associations between ${\mathrm{AUC}}_{\mathrm{ss},{\text{DOSE}}_{50},{\mathrm{CL}}_{\text{Cycle}1}}$ and the transition hazards underlying TTP and OS are summarized in **Table** **2**. In univariable analyses, a higher ${\mathrm{AUC}}_{\mathrm{ss},{\text{DOSE}}_{50},\text{cycle}1}$ value was associated with a lower hazard of progression from stable disease (λ_13_; *P* = 0.04). This effect was modest compared with the strong associations observed for baseline albumin (*P* < 0.001) and age (*P* < 0.001). After adjustments for these covariates, ${\mathrm{AUC}}_{\mathrm{ss},{\text{DOSE}}_{50},{\mathrm{CL}}_{\text{cycle}1}}$ was no longer associated with λ_13_ (*P* = 0.27). A trend toward a lower hazard of death after progression λ_36_ with higher ${\mathrm{AUC}}_{\mathrm{ss},{\text{DOSE}}_{50},{\mathrm{CL}}_{\text{cycle}1}}$ was also observed (*P* = 0.07), but this association was attenuated after adjustment for albumin (*P* = 0.41).

**Table 2:** Effect of ${\mathrm{AUC}}_{\mathrm{ss},{\text{DOSE}}_{50},{\mathrm{CL}}_{\text{cycle}1}}$ on transition hazards and time to first dose reduction

Model parameters	Description	Univariable analysis, ${\mathrm{HR}}_{\mathrm{IQR}}$ (95% CI), *P* value	Multivariable covariates adjusted	Multivariable analysis, ${\mathrm{HR}}_{\mathrm{IQR}}$ (95% CI), *P* value
λ_12_	Stable to Response	1.05 (0.88, 1.26), *P* = 0.42	–	–
λ_13_	Stable to Progression	0.71 (0.47, 1.07), *P* = 0.04	Age, albumin	1.13 (0.89, 1.45), *P* = 0.27
λ_14_	Stable to Disc. due to AEs	1.48 (0.93, 2.34), *P* = 0.02	–	–
λ_15_	Stable to Disc. due to other reasons	0.86 (0.45, 1.66), *P* = 0.92	–	–
λ_23_	Response to Progression	0.93 (0.70, 1.23), *P* = 0.82	–	–
λ_36_	Progression to Death	0.78 (0.59, 1.02), *P* = 0.07	Albumin	0.94 (0.50, 1.78), *P* = 0.41
λ	Time to first dose reduction	2.10 (1.62, 2.71), <0.001	–	–

${\mathrm{AUC}}_{\mathrm{ss},{\text{DOSE}}_{50},{\mathrm{CL}}_{\text{cycle}1}}$ was centered at the median and entered linearly on the log‐hazard scale: $h\left(t\right)=h_{0}\left(t\right)\cdot \exp\left(\beta \cdot \left(\mathrm{AUC}-2.19\right)\right)$. Reported hazard ratios correspond to an interquartile range (IQR) increase in AUC (Q75–Q25 = 2.99–1.62 = 1.37 μg·h/mL): ${\mathrm{HR}}_{\mathrm{IQR}}=\exp\left(\beta \cdot 1.37\right).$

Adjustment for certain covariates, indicating the exposure effect was evaluated after including the specified covariate(s) in the corresponding hazard parameter.

Rows λ_12_–λ_36_ are transition hazards from the multistate model; “Time to first dose reduction” was estimated from a separate parametric time‐to‐event model.

**Figure 5:**
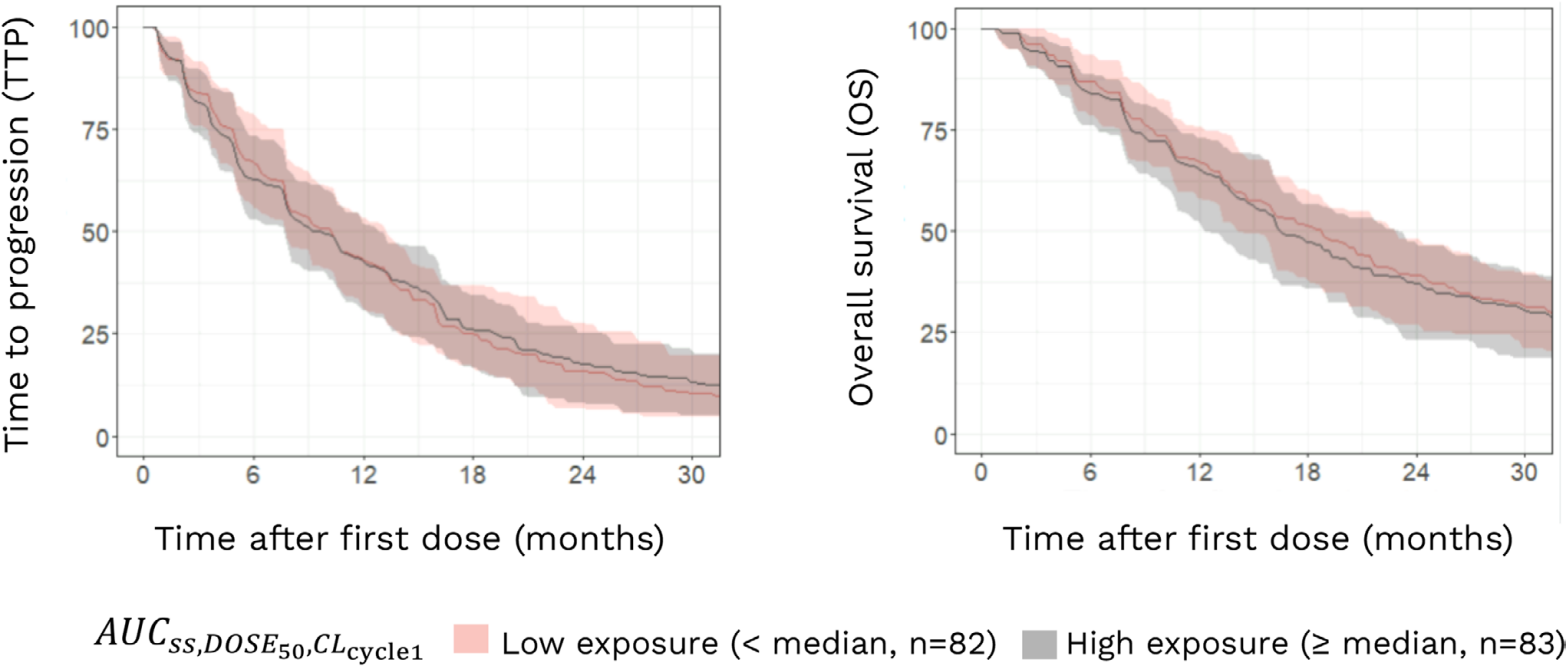
Confounding‐adjusted simulated time to progression (TTP) and overall survival (OS) by exposure group. TTP and OS were simulated from the final multivariable multistate survival model using the observed multistate dataset (*n* = 165) as the simulation template. To visualize expected outcomes under a counterfactual scenario with a comparable baseline prognosis, each patient's baseline albumin and age values were replaced with the cohort median values prior to simulation. Patients were stratified by the median ${\mathrm{AUC}}_{\mathrm{ss},{\text{DOSE}}_{50},{\mathrm{CL}}_{\text{cycle}1}}$. The solid lines represent the median, and the shaded areas indicate the 90% simulation interval based on 100 simulated replicates.

Simulated survival curves stratified by the median ${\mathrm{AUC}}_{\mathrm{ss},{\text{DOSE}}_{50},{\mathrm{CL}}_{\text{cycle}1}}$ overlapped for both TTP and OS across 100 replicates after baseline albumin and age were fixed to the cohort median for all patients (**Figure** **5**). These confounder‐fixed simulations indicated no meaningful difference in expected outcomes between low‐ and high‐exposure groups under a common baseline prognostic profile.

After bias adjustment, no association was identified between the alternative exposure metrics, including $C_{\text{trough},\mathrm{ss}}$ and unbound exposure, and clinical outcomes; the results were consistent with those obtained using the primary exposure metric. Consistent findings were also obtained in a supportive sensitivity analysis using the broader dataset, including both treatment‐naïve and cytokine‐refractory patients (*n* = 294), in which no independent positive exposure–efficacy association was identified.

### Exposure and tolerability‐related outcomes

In the univariate analysis, higher sunitinib exposure was associated with poorer tolerability. For each 1.37 μg·h/mL (interquartile range, IQR) increase in ${\mathrm{AUC}}_{\mathrm{ss},{\text{DOSE}}_{50},{\mathrm{CL}}_{\text{cycle}1}}$, the hazard of transition from stable disease to treatment discontinuation due to AEs (λ_14_) increased 1.48‐fold, and the hazard of dose reduction increased 2.10‐fold (**Table** **2**).

## DISCUSSION

This study uses sunitinib in mRCC as a case study to show that exposure–efficacy assessments of anticancer TKIs can be susceptible to both time‐dependent bias and confounding. After accounting for these sources of bias, cycle 1 systemic exposure was not independently associated with TTP or OS. These findings challenge the longstanding assumption that, among patients treated with the standard 50 mg 4/2 regimen, higher exposure is associated with superior efficacy. Instead, our results suggest that dose escalation based solely on systemic exposure is unlikely to improve clinical outcomes. By contrast, patients with higher early exposure experienced poorer tolerability, including more frequent dose reductions and a higher risk of treatment discontinuation due to AEs. Taken together, these findings support a strategy of dose de‐escalation in patients with excessive exposure rather than escalation in patients with lower exposure.

Our findings extend prior methodological work showing that conventional E–R analyses based on time‐averaged exposure can be systematically biased by post‐baseline processes, such as exposure accumulation and dose‐modification patterns.^15^ In this sunitinib example, the apparent beneficial association between higher exposure and both TTP and OS was attenuated when exposure was defined using the initial 50 mg dose and cycle 1 apparent clearance rather than time‐averaged apparent clearance. Specifically, the log‐rank *P*‐values increased from 0.032 to 0.15 for TTP and from 0.061 to 0.52 for OS, indicating a clear weakening of the observed associations. This pattern is consistent with declining apparent clearance over time, whereby patients who remain on treatment longer will, by construction, have higher time‐averaged exposure, thereby generating an artificial positive association between exposure and survival. Conversely, when exposure was defined using time‐averaged dose together with cycle 1 apparent clearance, the direction of association reversed, with higher exposure appearing to predict worse outcomes. This reversal is consistent with dose reductions occurring more frequently among patients who remain on therapy longer, thereby lowering the average dose and, consequently, the average exposure in those with a more favorable prognosis. Collectively, these findings show that both the magnitude and direction of the apparent E–R relationship for sunitinib depend strongly on how exposure is summarized. They also support the use of early, baseline‐anchored exposure metrics as a pragmatic approach to reduce time‐dependent bias in observational E–R analyses.^6^, ^10^, ^15^

After addressing time‐dependent bias, a weak positive association remained between higher cycle 1 exposure and longer TTP and OS, but this association disappeared after adjustment for baseline age and albumin. Older patients had lower apparent clearance of both sunitinib and SU12662 and therefore higher exposure, consistent with previous reports.^33^, ^43^ In univariable analyses, age was a stronger predictor of progression risk than systemic exposure itself, with older age associated with longer TTP. This suggests that the observed exposure–efficacy association was driven, at least in part, by more favorable baseline prognosis in older patients rather than by a causal benefit of higher exposure. Similarly, low baseline albumin, an established adverse prognostic factor in mRCC,^44^ was associated with higher apparent clearance, faster progression, and shorter survival after progression. Thus, patients with low albumin were both underexposed and predisposed to poor outcomes, with prognosis likely driven primarily by underlying disease biology, inflammation, and nutritional status rather than by drug exposure per se.

The association between albumin and apparent clearance is also biologically plausible. Sunitinib and SU12662 are highly protein‐bound in plasma (95% and 90%, respectively), primarily to albumin.^21^ Hypoalbuminemia would be expected to reduce binding, increase the unbound fraction, and lower total plasma concentrations, even if the pharmacologically active unbound concentrations remain relatively unchanged. When PK parameters are estimated from total concentrations, this phenomenon manifests as an increase in apparent clearance of total drug with lower albumin levels.^45^, ^46^, ^47^ Preclinical rat data support this mechanism, showing that lower albumin levels increase apparent clearance and reduce total sunitinib exposure without altering unbound concentrations.^48^ In line with this, we observed higher apparent clearance and lower total exposure in patients with lower baseline albumin, although unbound drug concentrations were not available in the present analysis. These observations indicate that E–R analyses based solely on total concentrations may be particularly vulnerable to bias when protein binding is altered. Unbound concentrations, although analytically more challenging to measure,^49^ may therefore provide a more mechanistically relevant and less confounded measure of active exposure.

In addition, changes in albumin or other disease‐related factors during treatment could, in theory, contribute to the observed decline in apparent clearance and apparent volume of distribution over time. However, the present data did not provide sufficient evidence to determine whether the individual magnitude of clearance decline tracked tumor response or reflected an exogenous pharmacokinetic time trend, given the high η‐shrinkage and limited longitudinal PK information. These results also have direct implications for the TDM practice of sunitinib. The presence of bias substantially weakens the rationale for previously proposed trough concentration targets for concentration‐guided dose escalation. In particular, the widely cited combined steady‐state trough thresholds of 50 and 60.75 ng/mL for sunitinib plus SU12662^38^, ^39^, ^40^ were derived from univariate positive E–R associations that did not account for time‐increasing exposure or confounding. On this basis, these thresholds should not be used to guide dose escalation in clinical practice. In other terms, our results do not support the definition of a target exposure threshold required for efficacy within the standard 50 mg 4/2 regimen based on current data, as no independent positive exposure–efficacy association was identified, despite substantial interindividual PK variability. Instead, TDM may be more appropriately applied to identify patients with high early‐cycle exposure who are at increased risk of toxicity and may benefit from dose reduction while remaining within an exposure range consistent with preserved efficacy.

Although our analyses adjusted for a broad set of clinically relevant baseline covariates, residual or unmeasured confounding cannot be excluded. This limitation is inherent to E–R analyses, particularly when all patients receive the same nominal dose, as in the present dataset. These findings therefore reinforce the importance of prospective dose‐ranging or dose‐randomized studies for TKIs, in line with the increasing emphasis on dose optimization in contemporary oncology drug development.^3^ In addition, the absence of an independently significant exposure–efficacy association should not be interpreted as definitive proof of a flat E–R relationship. Use of early, baseline‐anchored exposure metrics may reduce time‐dependent bias but can also reduce power to detect a true exposure effect.^8^ Therefore, the present findings are best interpreted as showing no evidence of an independent positive exposure–efficacy relationship within the observed exposure range after adjustment for bias and confounding, rather than excluding all possible exposure effects.

In conclusion, this case study demonstrates that both time‐dependent bias and biological confounding can distort E–R analyses of TKIs. In sunitinib‐treated mRCC, once these biases were addressed, higher early exposure was not independently associated with improved efficacy, whereas higher exposure remained associated with greater toxicity. These findings argue against exposure‐driven dose escalation based on currently proposed targets for the standard dose of 50 mg 4/2 and instead support a more cautious, bias‐aware use of pharmacometric analyses to inform dose optimization and TDM strategies in oncology.

## FUNDING

This work was supported by the Swedish Cancer Society (23 2921 Pj).

## CONFLICT OF INTEREST

The authors declared no competing interests for this work.

## AUTHOR CONTRIBUTIONS

H.L. and L.E.F. wrote the manuscript. H.L. and L.E.F. designed the research. H.L. performed the research and analyzed the data.

## Supporting information


Supplementary Material S1.

Supplementary Material S2.

Supplementary Material S3.
