# Supplementary material for: Time‐Dependent Bias and Prognostic Confounding in Exposure–Response Analysis of Targeted Therapies: Lessons from Sunitinib in Metastatic Renal Cancer
Source: Clin Pharmacol Ther. 2026 Aug 12:10.1002/cpt.70447. Online ahead of print. doi: 10.1002/cpt.70447 (PMC13468949; doi:10.1002/cpt.70447)
Supplement: Supplementary file 1 [file CPT-9999-0-s001.docx]

Supplementary material 3: Multistate modeling

**Software and Model Evaluation**

Model development was conducted in NONMEM (version 7.5) using the importance sampling (IMP) algorithm. Parameter uncertainty was quantified using the variance–covariance matrix of the estimated parameters. Model selection was based on likelihood-ratio tests, evaluated via changes in the OFV, and simulation-based diagnostics, including VPCs. Data preparation and model diagnostics were performed in R (version 4.0.4) and Xpose4 (version 4.7.1). Covariate analysis was performed using SCM with forward inclusion (p < 0.05) and backward elimination (p < 0.01) guided by the OFVs.

**Multistate Model Structure**

The state and transition diagrams are shown in **Figure 1** in the main manuscript**.** The probability of occupying state n ($P_{n}$) for each state was modeled as the amount in each compartment, quantified by transition hazard/rate $\lambda_{ij}$ from state $i$ to state $j$ following equations:

${dP_{1}}/{dt}=- P_{1}\cdot(\lambda_{12}(t) + \lambda_{13}(t) + \lambda_{14}(t) + \lambda_{15}(t) + \lambda_{16}(t))$

${dP_{2}}/{dt}=P_{1} \cdot\lambda_{12}\left( t \right) - P_{2} \cdot\left( \lambda_{23}\left( t \right) + \lambda_{24}\left( t \right) + \lambda_{25}\left( t \right) + \lambda_{26}\left( t \right) \right)$

${dP_{3}}/{dt}=P_{1} \cdot\lambda_{13}\left( t \right) +P_{2}\cdot\lambda_{23}\left( t \right) - P_{3}\cdot\lambda_{36}\left( t \right)$

${dP_{4}}/{dt}=P_{1}\cdot\lambda_{14}\left( t \right) + P_{2} \cdot\lambda_{24}\left( t \right) - P_{4} \cdot\lambda_{46}\left( t \right)$

$${dP_{5}}/{dt}=P_{1} \cdot\lambda_{15}\left( t \right) + P_{2} \cdot\lambda_{25}\left( t \right) - P_{5} \cdot\lambda_{56}\left( t \right)$$

$${dP_{6}}/{dt}=P_{1} \cdot\lambda_{16}\left( t \right) + P_{2} \cdot\lambda_{26}\left( t \right) + P_{3}\cdot\lambda_{36}\left( t \right) + P_{4} \cdot\lambda_{46}\left( t \right)+ P_{5} \cdot\lambda_{56}\left( t \right)$$

**Multistate Model Assumptions**

It was assumed that all patients were in a stable state (S1) on the date of treatment initiation. Transitions were specified as unidirectional to reflect the clinical irreversibility of disease progression and treatment discontinuation per study protocol. Patients were censored in their current state at study completion.

**Derivation of Clinical Outcomes**

Clinical efficacy endpoints, time to progression (TTP), overall survival (OS), and time to discontinuation due to AEs, were derived from the multistate model:

- **TTP** was defined as the time from treatment initiation to progression (S3). Patients who died directly from stable disease (S1) or response (S2) were censored at the date of death. Patients who discontinued due to AEs (S4), discontinued for other reasons (S5), or were right-censored at the end of follow-up without prior progression were censored at the date of the last available tumor evaluation. If no post-treatment tumor assessment was available and the patient did not die, TTP was censored on day 1.
- **OS** was defined as the time from treatment initiation to death (S6), and was right-censored at the last observed state for surviving patients.
- **Time to discontinuation due to AEs** was defined as the time from treatment initiation to discontinuation for AEs (S4). Patients remaining in S1 or S2 were censored at the end of follow-up, while those who transitioned to progression (S3), discontinuation for other reasons (S5), or death (S6) were censored at the date of that event.

**Hazard Estimation and Covariate Analysis**

Both constant and time-dependent hazard functions were evaluated. A constant hazard was represented by an exponential distribution, whereas time-varying hazards were described using Weibull models parameterized by time since treatment initiation, time in the previous state, or time spent in the current state. Alternative parametric forms, including log-logistic and Gompertz distributions, were also explored.

Exponential function: $\lambda_{ij}={scale}_{ij}$

Weibull function: $\lambda_{ij}\left( t \right)={scale}_{ij}\cdot{shape}_{ij}\cdot\left( {scale}_{ij}\cdot t \right)^{{shape}_{ij}-1}$

Covariates were tested as predictors for all estimated transitions. For individual $z$ with continuous covariate $X$, the transition hazards will be:

$${\lambda_{ij, k}=\lambda}_{ij,0}\cdot e^{\left( {\beta_{X} \cdot(X}_{z}-X_{median}) \right)}$$

Where $k_{ij,0}$ is the baseline transition hazard, $\beta_{X}$ is the coefficient of the effect of X on the transition from state i to j. $X_{median}$ is the population median value of X for baseline covariates, and the median value of X at baseline for time-varying covariates. A study-specific categorical covariate was tested on all parameters to account for potential between-study heterogeneity.

In the final multistate model, most transition hazards were time-constant, except for $\lambda_{12}$ (stable disease to response), which decreased over time from the first dose, following a Weibull distribution, and $\lambda_{36}$ (progression to death), which decreased with longer time to progression, described using a log-logistic function.

For sparsely observed transitions, model complexity was limited by fixing or merging transition hazards where appropriate, and no covariate effects were retained for these transitions. Due to a limited number of observed transitions from stability to death (n=2) and response to death (n=4), $\lambda_{16}$ and $\lambda_{26}$ were estimated with high uncertainty and subsequently fixed to the age-specific natural death rates estimated using the Gompertz-Makeham distribution ^1^. Notably, the OFV did not increase significantly after fixing these two parameters compared with the model in which they were estimated. $\lambda_{15}$ and $\lambda_{25}$, describing transitions from stable disease or response to discontinuation due to other reasons, were estimated to have similar values and were therefore merged and described by a common parameter. This did not result in a statistically significant increase in OFV. The same approach was applied to $\lambda_{46}$ and $\lambda_{56}$, describing transitions from discontinuation due to adverse events or other reasons to death. Note that covariate effects were not retained for these sparse transitions; therefore, the limited observations mainly affects the interpretation of the corresponding baseline hazards rather than the reported covariate findings.

**Table S4.** Parameter Estimates and Uncertainty of the Final Multistate Model.

| **Category** | **Parameter** | **Transition** | **Functions** | **Estimate ^a^** | **RSE (%)** |  |
| --- | --- | --- | --- | --- | --- | --- |
| Baseline transitions (week⁻¹) ^b^ | $\lambda_{12}$ scale | Stable → Response | Weibull scale | **0.0471** | **13.4** |  |
|  | Time since treatment initiation on $\lambda_{12}$ | Stable → Response | Weibull shape | **0.844** | **11.5** |  |
|  | $\lambda_{13}$ | Stable → Prog. | Exponential | **0.00725** | **30.5** |  |
|  | $\lambda_{23}$ | Response → Prog. | Exponential | **0.0171** | **13.5** |  |
|  | $\lambda_{14}$ | Stable → Disc. AEs | Exponential | **0.00758** | **25.7** |  |
|  | $\lambda_{24}$ | Response → Disc. AEs | Exponential | **0.000758** | **57.7** |  |
|  | $\lambda_{15}$ ^c^ | Stable → Disc. other | Exponential | **0.00131** | **37.2** |  |
|  | $\lambda_{25}$ ^c^ | Response → Disc. other | Exponential |  |  |  |
|  | $\lambda_{16}$ | Stable → Death | Gompertz–Makeham ^d^ | fixed |  |  |
|  | $\lambda_{26}$ | Response → Death | Gompertz–Makeham ^d^ | fixed |  |  |
|  | $\lambda_{36}$ | Prog. → Death | Log-logistic | **0.0340** | **39.5** |  |
|  | Time to Prog. on $\lambda_{36}$ | Prog. → Death | Log-logistic | **0.930** | **2.8** |  |
|  | $\lambda_{46}$ ^e^ | Disc. AEs → Death | Exponential | **0.0267** | **22.4** |  |
|  | $\lambda_{56}$ ^e^ | Disc. other → Death | Exponential |  |  |  |
| Covariate effects | ALB on $\lambda_{13}$ | Stable → Prog. | log-HR: exp((ALB−41)·θ) | **−0.165** | **12.4** |  |
|  | HB on $\lambda_{13}$ | Stable → Prog. | log-HR: exp((HB−130)·θ) | **−0.0255** | **32.4** |  |
|  | AGE on $\lambda_{13}$ | Stable → Prog. | log-HR: exp((AGE−57)·θ) | **−0.0892** | **13.9** |  |
|  | ECOG on $\lambda_{13}$ | Stable → Prog. | HR: ECOG ≥1 vs 0 | **1.48** | **29.3** |  |
|  | TDIAG on $\lambda_{13}$ | Stable → Prog. | HR: TDIAG >1y vs ≤1y | **0.950** | **31.8** |  |
|  | ALB on $\lambda_{36}$ | Prog. → Death | log-HR: exp((ALB−41)·θ) | **−0.0731** | **39.5** |  |
|  | TDIAG on $\lambda_{36}$ | Prog. → Death | HR: TDIAG >1y vs ≤1y | **0.678** | **26.7** |  |
| Abbreviations: Prog.=progression; Disc. = discontinuation; AEs=adverse events; TDIAG=time since diagnosis; SLD=sum of longest diameters of target lesions at baseline (mm). CI=confidence interval.   a. Parameter uncertainty obtained from the covariate step with the R matrix. b. Baseline hazards correspond to HB=130 g/L, ALB=41 g/L, AGE=57 years, ECOG=0, TDIAG≤1 year. c. Merged in estimation. d. Fixed to Gompertz–Makeham distribution ^1^. $h_{GM}(age) = 0.0007+0.00018645 \cdot e^{0.11425(age-30)} {year}^{-1}$ e. Merged in estimation. | | | | |  | |

## References

1.	Missov, T. I. & Lenart, A. Gompertz–Makeham life expectancies: Expressions and applications. *Theor. Popul. Biol.* **90**, 29–35 (2013).
