# Supplementary material for: Time‐Dependent Bias and Prognostic Confounding in Exposure–Response Analysis of Targeted Therapies: Lessons from Sunitinib in Metastatic Renal Cancer
Source: Clin Pharmacol Ther. 2026 Aug 12:10.1002/cpt.70447. Online ahead of print. doi: 10.1002/cpt.70447 (PMC13468949; doi:10.1002/cpt.70447)
Supplement: Supplementary file 2 [file CPT-9999-0-s002.docx]

Supplementary material 2: Pharmacokinetic data and modelling

**Model Estimation and Evaluation**

Model development was conducted in NONMEM (version 7.5) using the importance sampling (IMP) algorithm. Parameter uncertainty was quantified using sampling importance resampling (SIR)^1^. Model selection was based on likelihood ratio tests, evaluated through changes in the objective function value (OFV), and simulation-based diagnostics, including goodness-of-fit (GOF) plots and visual predictive checks (VPCs). Data preparation and model diagnostics were performed in R (version 4.0.4) and Xpose4 (version 4.7.1). Covariate analysis was performed using stepwise covariate modeling (SCM) with forward inclusion (p < 0.05) and backward elimination (p < 0.01) guided by the OFVs.

**Analysis Dataset**

The final dataset included 1,688 sunitinib and 1,715 SU12662 concentrations from 294 patients, after excluding 55 values below the quantification limit (Figure S1).

**Population PK Model Structure**

Interindividual variability (IIV) was modeled using exponential functions and estimated for apparent clearance ($CL/F$), apparent central ($V_{c}/F$) and peripheral ($V_{p}/F$) volumes of distribution for both sunitinib and SU12662, as well as for bioavailability ($F$) and fraction of sunitinib metabolized to SU12662 ($f_{m}$). Correlations were incorporated between individual CL/F estimates of the parent drug and metabolite, and between their respective $V_{c}/F$ and $V_{p}/F$ estimates. Residual variability was described using a proportional error model.

**Time-varying CL/F and V/F**

To account for temporal changes in pharmacokinetics, $CL/F$ and $V/F$ for both sunitinib and SU12662 were modelled as time-varying:

$$CL/F =CL_{0}/F \cdot\frac{1}{1+\beta_{CL}\cdot T}$$

$V_{c}/F =V_{c0}/F \cdot\frac{1}{1+\beta_{V}\cdot T}$ $V_{p}/F =V_{p0}/F \cdot\frac{1}{1+\beta_{V}\cdot T}$

where $CL_{0}/F$, $V_{c0}/F$ and $V_{p0}/F$ denote the baseline values$, T$ is time in weeks, and $\beta$ is the rate parameter describing the decline.

IIV on $\beta$ was modelled additively to allow both time-increasing and decreasing individual trends. Two random effects were included: one for clearance ($\eta_{CL}$) and one for volume of distribution ($\eta_{V}$). These $\eta$s were shared between sunitinib and SU12662 to account for the correlation between the parent and metabolite, while estimated scaling factors determined their relative contributions:

$$\beta_{CL,sunitinib\boldsymbol{, i}}= \beta_{CL,sunitinib}+ \eta_{CL,i}$$

$$\beta_{CL,SU12662\boldsymbol{, i}}= \beta_{CL,SU12662}+ \eta_{CL,i}/\theta_{CL, SU12662}$$

$$\beta_{V,sunitinib\boldsymbol{, i}}= \beta_{V,sunitinib}+ \eta_{V,i}$$

$$\beta_{V,SU12662\boldsymbol{, i}}= \beta_{V,SU12662}+ \eta_{V,i}/\theta_{V, SU12662}$$

In this way, a single $\eta$ captured IIV in clearance or volume for both analytes, while the scaling factors allowed flexibility in the magnitude of the effects across the two compounds.

Alternative functional forms, including exponential and Tmax functions, were also explored to characterize time-varying apparent clearance, but did not improve the objective function value relative to the final model.

**Model Diagnostics**

GOF plots (Figure S3) for the final population PK model, including the time-varying structure, demonstrated an adequate description of both sunitinib and SU12662 concentrations across the observed range. Conditional weighted residuals were symmetrically distributed around zero, with no systematic trends over time or with predicted concentrations, indicating no major model misspecification.

VPCs (Figure S4) further supported the adequacy of the final model. A comparison of VPCs from the model without time-varying PK versus the model with time-varying PK highlighted the latter's improved ability to capture the observed temporal decline in apparent clearance and volume of distribution. In particular, the inclusion of time-varying effects reduced the systematic underprediction of concentrations at later time points and improved the alignment between observed and simulated percentiles.

Taken together, the GOF plots and VPCs confirmed that the time-varying PK model provided a more reliable description of the data compared with a model assuming constant apparent clearance and volume over time.

**Table S2. Parameter estimates of the final PK model**

|  | **Parent** | | **Metabolite** | |
| --- | --- | --- | --- | --- |
|  | **Estimate** | **RSE(%)** | **Estimate** | **RSE(%)** |
| Ka (1/h) | 0.224 (fixed) | - | 0.132 (fixed) | - |
| $\mathrm{CL}_{0}/F$(L/h) | 35.3 | 3.2% | 18.1 | 2.7% |
| ${Vc}_{0}/F$ (L) | 2250 | 2.9% | 2210 | 4.2% |
| Q$/F$ (L/h) | 1.72 | 10.9% | 1.02 | 14.7% |
| ${Vp}_{0}/F$ (L) | 272 | 6.1% | 384 | 15.8% |
| $F_{sunitinib}$ ^a^ | 1 (fixed) | - | - | - |
| $f_{m}$ ^a^ | - | - | 0.21 (fixed) | - |
| SEX on $F_{sunitinib}$ ^b^ | 0.148 | 10.9% | - | - |
| SEX on $f_{m}$ ^b^ | - | ¨ | 0.243 | 18.6% |
| SEX on CL/F ^b^ | –0.0985 | 12.7% | –0.0864 | 17.4% |
| AGE on CL/F ^c^ | –0.00590 | 15.1% | –0.00793 | 14.2% |
| ALB on CL/F ^d^ | –0.507 | 19.0% | –0.507 | 19.0% |
| ALB on Vc/F ^d^ | –0.741 | 12.9% | –0.741 | 12.9% |
| WT on CL/F ^e^ | - | - | 0.217 | 14.9% |
| WT on Vc/F ^e^ | 0.220 | 19.9% | 0.447 | 14.9% |
| $\beta_{TIME, CL}$ ^f^ | 0.00365 | 13.3% | 0.00801 | 11.6% |
| $\beta_{TIME, Vc}$^f^ | 0.00168 | 40.5% | 0.0125 | 20.7% |
| **Exponential IIV (%CV^g^)** |  |  |  |  |
| IIV $F_{sunitinib}$ | 26.5 | 5.9% | - | - |
| IIV $f_{m}$ | - | - | 22.6 | 8.5% |
| IIV CL/F | 9.91 | 19.1% | 15.1 | 8.7% |
| IIV Vc/F | 8.30 | 21.5% | 11.7 | 15.1% |
| IIV Vp/F | 26.1 | 19.3% | 59.4 | 22.9% |
| **Additive IIV and scales^h^** |  |  |  |  |
| IIV $\beta_{TIME, CL/F}$ (%CV^g^) | 0.8 | 10.3% | 0.8 | 10.3% |
| IIV $\beta_{TIME, CL/F}$ scale | - | - | 0.90 | 24.0% |
| IIV $\beta_{TIME, Vc/F}$ (%CV^g^) | 0.9 | 21.1% | 0.9 | 21.1% |
| IIV $\beta_{TIME, Vc/F}$ scale | - | - | 1.38 | 25.6% |
| **Correlation of IIV** |  |  |  |  |
| Correlation CL$/F$ (Parent–Met) | 0.70 | 15.5% | - | - |
| Correlation V$/F$ (Parent–Met) | 0.68 | 21.0% | - | - |
| **RUV (% CV^g^)** |  |  |  |  |
| Residual unexplained variability | 21.7% | 1.7% | 21.9% | 2.1% |
| Abbreviations: Ka, absorption rate; CL, clearance; F, bioavailability; Vc, central volume distribution; Vp, peripheral volume distribution; Q, inter-compartment clearance; $f_{m}$, fraction metabolized; CV, coefficient of variation; ALB, albumin; WT, body weight. | | | | |
| a. Bioavailability for SU12662 $\boldsymbol{F}_{\boldsymbol{SU}\boldsymbol{12662}}$= $\boldsymbol{F}_{\boldsymbol{sunitinib}}\boldsymbol{\cdot}\boldsymbol{f}_{\boldsymbol{m}}$ b. Sex was coded as an indicator variable (SEX = 0 for males, 1 for females) and implemented as a multiplicative factor 1 + $\boldsymbol{\theta}_{\boldsymbol{SEX}}\boldsymbol{\cdot}\boldsymbol{SEX}$ c. Exponential function centered around the median age $\boldsymbol{e}^{\mathbf{(}\mathbf{AGE}\mathbf{-}\mathbf{60}\mathbf{)*}\boldsymbol{\theta}_{\boldsymbol{AGE}}}$ d. Power function centered around the median albumin ${\boldsymbol{(}\boldsymbol{ALB}\boldsymbol{/}\boldsymbol{41}\boldsymbol{)}}^{\boldsymbol{\theta}_{\boldsymbol{ALB}}}$ e. Power function centered around the median weight ${\boldsymbol{(}\boldsymbol{WT}\boldsymbol{/}\boldsymbol{82}\boldsymbol{.}\boldsymbol{4}\boldsymbol{)}}^{\boldsymbol{\theta}_{\boldsymbol{WT}}}$ f. Time-varying PK parameters were coded as baseline value multiplied by 1/(1+$\boldsymbol{\beta}_{\boldsymbol{TIME}}$*Week) g. Reported as the square root of estimated variances $\boldsymbol{\times}\boldsymbol{100}\boldsymbol{\%}$ h. Individual $\boldsymbol{\beta}_{\boldsymbol{TIME}\boldsymbol{,}\boldsymbol{i}}\boldsymbol{=}\boldsymbol{\beta}_{\boldsymbol{TIME}}\boldsymbol{+}\boldsymbol{\eta}_{\boldsymbol{i}}\boldsymbol{/}\boldsymbol{scale}$ | | | | |

**Table S3.** Descriptive statistics of exposure metrics (units: µg·h/mL) across individuals in the exposure-response dataset (n=165).

| **Exposure metric** | **Analyte** | **Median (IQR)** | **Min–Max** |
| --- | --- | --- | --- |
| $\boldsymbol{AUC}_{\boldsymbol{ss}\mathbf{,}\boldsymbol{DOSE}_{\boldsymbol{50}}\mathbf{,}\boldsymbol{CL}_{\boldsymbol{ave}}}$ | Sunitinib | 1.56 (1.18,2.08) | 0.44-3.56 |
|  | SU12662 | 0.75 (0.53, 1.05) | 0.16-2.77 |
|  | Total | 2.27 (1.76, 2.98) | 0.61-5.97 |
| $\boldsymbol{AUC}_{\boldsymbol{ss}\mathbf{,}\boldsymbol{DOSE}_{\mathbf{50}}\mathbf{,}\boldsymbol{CL}_{\boldsymbol{cycle}\boldsymbol{1}}}$ | Sunitinib | 1.54 (1.13,1.98) | 0.40-3.54 |
|  | SU12662 | 0.67 (0.49, 0.98) | 0.15-2.54 |
|  | Total | 2.19 (1.62, 2.99) | 0.56-5.20 |
| $\boldsymbol{AUC}_{\boldsymbol{ss}\mathbf{,}\boldsymbol{DOSE}_{\boldsymbol{ave}}\mathbf{,}\boldsymbol{CL}_{\boldsymbol{cycle}\boldsymbol{1}}}$ | Sunitinib | 0.94 (0.74, 1.32) | 0.30-2.78 |
|  | SU12662 | 0.45 (0.31, 0.63) | 0.15-1.41 |
|  | Total | 1.38 (1.09, 1.98) | 0.47-4.11 |

**Figure S2.** Observed plasma concentration

ns of sunitinib (A) and its active metabolite SU12662 (B) over time after the first dose, stratified by clinical study (014, 034, 065, 1006). Individual points represent measured plasma concentrations (ng/mL) plotted on a semi-logarithmic scale against time since the first dose (weeks). The lower limit of quantification was 0.1 ng/mL for studies 014 and 1006, and 1 ng/mL for studies 034 and 065.

**Figure S3.** Goodness-of-fit plots for the final population PK model of sunitinib and SU12662. Panels show observed versus population-predicted concentrations (top left), observed versus individual-predicted concentrations (top right), conditional weighted residuals (CWRES) versus time after first dose (bottom left), and CWRES versus population predictions (bottom right). The solid red line represents the line of identity (top panels) or the zero line (bottom panels), and the solid blue line is a linear fit to the data.

**Figure S4.** Visual predictive checks (VPCs) for sunitinib and SU12662, comparing models with and without time-varying pharmacokinetics. Observed median and 10th/90th percentiles (solid lines) are shown with 95% prediction intervals from 500 simulations (shaded areas). The model without time-varying PK systematically underpredicted concentrations at later time points, whereas the final model with time-varying PK reproduced the observed increase in concentrations over time.

**Figure S5. Distribution of exposure metrics at steady state for the 50-mg dose in cycle 1.** Histograms show the distribution of ${AUC}_{ss, {DOSE}_{50}, {CL}_{cycle1}}$ (µg·h/mL) and (right) $C_{trough,ss, {DOSE}_{50}, {CL}_{cycle1}}$ (ng/mL) across individuals in the analysis dataset (n=165). Both are calculated based on individual empirical Bayes estimates (EBEs) of individual PK parameters at cycle 1.

**Figure S6.** Correlation between steady-state trough concentration ($C_{trough,ss,DOSE 50, {CL}_{cycle1}}$) and steady-state area under the concentration–time curve (${AUC}_{ss, {DOSE}_{50}, {CL}_{cycle1}}$) calculated based on the initial dose of 50 mg and individual PK parameters based on cycle 1 PK data across individuals in the population pharmacokinetic analysis dataset (n=294). The Pearson correlation coefficient (r = 0.99) is indicated on the plot.

**Figure S7.** Correlation between individual-level ${AUC}_{ss, {DOSE}_{50}, {CL}_{cycle1}}$ for sunitinib (x-axis) and SU12662 (y-axis) across individuals in the population pharmacokinetic analysis dataset (n=294).

## References

1.	Missov, T. I. & Lenart, A. Gompertz–Makeham life expectancies: Expressions and applications. *Theor. Popul. Biol.* **90**, 29–35 (2013).
