# Supplementary material for: Time‐Dependent Bias and Prognostic Confounding in Exposure–Response Analysis of Targeted Therapies: Lessons from Sunitinib in Metastatic Renal Cancer
Source: Clin Pharmacol Ther. 2026 Aug 12:10.1002/cpt.70447. Online ahead of print. doi: 10.1002/cpt.70447 (PMC13468949; doi:10.1002/cpt.70447)
Supplement: Supplementary file 3 [file CPT-9999-0-s003.docx]

Supplementary material 1: Baseline patient characteristics

**Table S1**: Baseline characteristics of the E–R analysis set (n=165), presented as mean (standard deviation) [minimum; maximum] {number of subjects missing} or number of subjects (%)

| **Baseline characteristics** | **Summary statistics** |
| --- | --- |
| Age (years) | 57.1 (10.3)  [24.0 ; 87.0] |
| Body weight (kg) | 85.0 (18.6) [45.0 ; 150.0] {2} |
| Sex   - Female - Male | 58 (35%)  107 (65%) |
| Race   - White - Non-white | 151 (92%)  14 (8%) |
| Albumin (g/L) | 40.4 (4.8) [20.0 ; 49.0] |
| Haemoglobin (g/L) | 124.3 (18.8) [86.0 ; 169.0] {13} |
| Calcium (mmol/L) | 2.3 (0.2) [1.9 ; 3.2] {1} |
| Sum of the longest diameters of targeted lesions RECISIT 1.0 (mm) | 136.4 (85.1) [12.0 ; 481.0] {3} |
| Time since diagnosis (months) | 38.0 (53.6)  [2.2 ; 339] |
| ECOG score   - 0 - 1 - >1 | 91 (55%)  72 (44%)  2 (1%) |
| Liver metastases   - Yes - No | 38 (23%)  127 (77%) |

**Figure S1.** Correlation matrix of baseline covariates for the 165 individuals included in the exposure–efficacy analysis. Numbers in each cell represent Pearson’s correlation coefficient (r). The color scale indicates the strength and direction of correlation (blue = positive, red = negative). Abbreviations: AGE, age; ALB, albumin; ALP, alkaline phosphatase; ALT, alanine aminotransferase; ANC, absolute neutrophil count; AST, aspartate aminotransferase; BMI, body mass index; CLcr, creatinine clearance; ECOG, Eastern Cooperative Oncology Group performance status; FFM, fat-free mass; HT, height; LDH, lactate dehydrogenase; RACE, race; SEX, sex; SLD, sum of longest diameters (target tumor size); Tdiag, time since diagnosis; WBC, white blood cell count; WT, body weight.
